# The *APOE* ε4 allele is associated with a reduction in FEV_1_/FVC in women: A cross-sectional analysis of the Long Life Family Study

**DOI:** 10.1371/journal.pone.0206873

**Published:** 2018-11-09

**Authors:** Alexander M. Kulminski, Amisha V. Barochia, Yury Loika, Nalini Raghavachari, Konstantin G. Arbeev, Mary K. Wojczynski, Bharat Thyagarajan, Badri N. Vardarajan, Kaare Christensen, Anatoliy I. Yashin, Stewart J. Levine

**Affiliations:** 1 Biodemography of Aging Research Unit, Social Sciences Research Institute, Duke University, Durham, NC, United States of America; 2 Laboratory of Asthma and Lung Inflammation, Pulmonary Branch, National Heart, Lung, and Blood Institute, Bethesda, MD, United States of America; 3 National Institute on Aging, Gateway Building, Suite, Bethesda, MD, United States of America; 4 Division of Statistical Genomics, Department of Genetics, School of Medicine, Washington University in St. Louis, St. Louis, Missouri, United States of America; 5 Department of Laboratory Medicine and Pathology, University of Minnesota, Minneapolis, MN, United States of America; 6 Taub Institute for Research on Alzheimer's Disease and the Aging Brain, Columbia University Medical Center, New York, NY, United States of America; 7 The Danish Aging Research Center, University of Southern Denmark, Odense C, Denmark; 8 Department of Clinical Genetics and Department of Clinical Biochemistry and Pharmacology, Odense University Hospital, Odense C, Denmark; Nathan S Kline Institute, UNITED STATES

## Abstract

**Introduction:**

Murine studies have shown that apolipoprotein E modulates pulmonary function during development, aging, and allergen-induced airway disease. It is not known whether the polymorphic human *APOE* gene influences pulmonary function.

**Objectives:**

We assessed whether an association exists between the polymorphic human *APOE* ε2, ε3, and ε4 alleles and pulmonary function among participants in the Long Life Family Study.

**Methods:**

Data from 4,468 Caucasian subjects who had genotyping performed for the *APOE* ε2, ε3, and ε4 alleles were analyzed, with and without stratification by sex. Statistical models were fitted considering the effects of the ε2 allele, defined as ε2/2 or ε2/3 genotypes, and the ε4 allele, defined as ε3/4 or ε4/4 genotypes, which were compared to the ε3/3 genotype.

**Results:**

The mean FEV_1_/FVC ratio (the forced expiratory volume in one second divided by the forced vital capacity) was lower among women with the ε4 allele as compared to women with the ε3/3 genotype or the ε2 allele. Carriage of the *APOE* ε4 allele was associated with FEV_1_/FVC, which implied lower values. Further analysis showed that the association primarily reflected women without lung disease who were older than 70 years. The association was not mediated by lipid levels, smoking status, body mass index, or cardiovascular disease.

**Conclusions:**

This study for the first time identifies that the *APOE* gene is associated with modified lung physiology in women. This suggests that a link may exist between the *APOE* ε4 allele, female sex, and a reduction in the FEV_1_/FVC ratio in older individuals.

## Introduction

Apolipoprotein E (APOE) plays an essential role in normal lipid homeostasis by removing triglyceride- and cholesterol-containing lipoprotein particles from plasma by binding to cellular receptors of the LDL receptor (LDLR) family[1–3]. Consistent with this, individuals with familial APOE deficiency have severely disrupted lipid catabolism with type III hyperlipoproteinemia and premature cardiovascular disease[4]. In addition to lipid transport, APOE also has important anti-oxidant and anti-inflammatory properties[5–7].

The APOE protein is primarily synthesized by the liver and circulates in plasma[2, 3]. APOE is also expressed by macrophages, alveolar epithelial cells and pulmonary artery smooth muscle cells in the lung, where it may regulate respiratory health and disease[7–12]. For example, a role for APOE in normal lung development has been suggested as male *Apoe*-deficient mice have increased airway resistance in early adulthood and more rapid loss of lung recoil with age[13, 14]. This suggested that altered lipid metabolism in the absence of APOE may increase inflammation and oxidative stress with resultant abnormal lung mechanics. APOE may also modulate normal surfactant phospholipid synthesis by type II airway epithelial cells. *Apoe*-deficient mice have increased alveolar levels of the major surfactant lipid, disaturated phosphatidylcholine, which occurs as a consequence of elevated serum levels of triglycerides and very low-density lipoproteins (VLDL)[15]. Studies in murine models have shown that feeding *Apoe*-deficient mice a high-fat Western diet causes cholesterol accumulation in the lung with resultant pathogenic changes resembling emphysema, pulmonary fibrosis, pulmonary hypertension, and sarcoidosis[7, 16–21]. APOE also has been shown to have protective roles in murine models of asthma, acute lung injury (ALI), emphysema, and pulmonary hypertension[7, 10, 12, 22–25]. For example, APOE can directly bind and neutralize lipopolysaccharide from the cell walls of gram-negative bacteria, which may be a protective mechanism in ALI[26–29]. APOE can also bind mycobacterial lipid antigens, which are then internalized by antigen-presenting cells, which promotes adaptive immune responses to *Mycobacterium tuberculosis*[30].

It is not known whether the polymorphic human *APOE* gene influences pulmonary function. Here, we hypothesized that the common polymorphic *APOE* alleles, ε2, ε3 and ε4, might be associated with modified lung function among 4,554 participants of the Long Life Family Study (LLFS), which is a family-based cohort study that has the goal of identifying factors related to human longevity. We focused on spirometric measures of lung function, including the forced expiratory volume in one second (FEV_1_), the forced vital capacity (FVC), and the FEV_1_/FVC ratio (FEV_1_ divided by the FVC). These are standardized, universal measurements of air flow in and out of lungs that are routinely used, in both clinical care and research, to quantify and monitor obstructive lung diseases, such as asthma and chronic obstructive pulmonary disease (COPD), as well as restrictive lung disease, such as pulmonary fibrosis.

## Methods

### The Long-life family study

Baseline information from 4,954 U.S. and Danish LLFS participants was collected at four field centers using similar questionnaires and in-home physical examinations between 2006 and 2009 on families showing exceptional familial longevity ranked on the basis of a Family Longevity Selection Score, which is a summary-measure of the survival experience of the oldest living generation of siblings relative to what would be expected based on the life tables of a normal population. Disease information was assessed both retrospectively, from self-reports at baseline, and prospectively through 2015. Other eligibility criteria, as well as the design of the LLFS study have previously been described [31–35]. In brief, the U.S. families eligible for the LLFS study must have at least two living siblings aged 80+ years, two living offspring, and a living spouse of either siblings or offspring. Eligible families were enrolled in LLFS if at least three living members (a proband, at least one sibling, and one of their offspring) were willing to participate. In Denmark, individuals who would be aged 90+ years according the Danish National Register of Person were identified and contacted to further assess the family’s eligibility using criteria parallel to those used in the U.S.[34]. Thus, the LLFS includes long-living individuals (probands and siblings), their offspring, and spouses of probands and offspring.

Local institutional review boards of all participating centers approved the LLFS (University of Southern Denmark S-VF-2003227; Boston University H-25140; Columbia University AAAP4119; University of Pittsburgh IRB0509062 and PRO14050142) and all participants provided informed consent[36].

### Sample collection and processing

Baseline blood samples were collected from all LLFS participants and processed within 48 to 72 hours to isolate serum, plasma, DNA and cryopreserved lymphocytes. DNA was isolated from white blood cells using the automated Autopure DNA extraction method (Qiagen Inc., Gaithersburg, MD). For the 4% of participants from whom blood could not be obtained, DNA was isolated from buccal samples using Oragene kits (DNA Genotek, Ottawa, ON, Canada). The data presented in this manuscript utilized DNA from both sources. Lipid measurements were made from serum.

### *APOE* genotyping

Genotyping of the *APOE* ε2, ε3, and ε4 alleles for LLFS study participants was performed at the Biomedical Genomics Center at the University of Minnesota[37]. *APOE* genotyping was based on SNPs rs7412 and rs429358 using the Taqman genotyping platform and concordance among genotypes for both SNPs was 100% based on blinded duplicates. The frequencies of the *APOE* genotypes in a sample of 4,554 LLFS participants were ε2/ε2 (n = 32, 0.7%); ε2/ε3 (n = 683, 15.0%); ε2/ε4 (n = 86, 1.9%); ε3/ε3 (n = 2959, 65.0%); ε3/ε4 (n = 746, 16.4%); and ε4/ε4 (n = 48, 1.0%). The *APOE* genotypes were in Hardy-Weinberg equilibrium (p = 0.754), thereby implying that the observed genotype distribution did not deviate from the expected distribution in this population. Given the small number of carriers of the three uncommon genotypes, ε2/ε2, ε2/ε4, and ε4/ε4 (3.6% of the LLFS participants), we selected three groups for analysis. The ε2 allele was defined as ε2/ε2 or ε2/ε3 genotypes, the ε4 allele was defined as ε3/ε4 or ε4/ε4 genotypes, and the ε3/ε3 genotype was designated as the reference group for the association analyses using regression models (see **Analysis** section). Statistical models were fitted considering the effect of the ε2 and ε4 alleles as compared to the common ε3/ε3 reference genotype.

We prospectively determined that participants with the ε2/ε4 genotype would not be included in the models to avoid biasing the effects of the ε4 allele on pulmonary function by the ε2 allele. After exclusion of carriers of the *APOE* ε2/ε4 genotype, 4,468 Caucasian LLFS participants were analyzed. They included long-living (LL) individuals (N = 1,302), their offspring (N = 2,259), spouses of LL individuals (N = 158), and spouses of offspring (N = 749).

### Lung function outcomes

Quantitative markers of lung function, FEV_1_, FVC, and FEV_1_/FVC (%), were considered as outcomes. Lung function was measured with a portable spirometer (EasyOne, ndd Medical Technologies, Andover, MA) using American Thoracic Society guidelines[38].

### Analysis

In the initial analyses, mean values (age, BMI, FEV_1_, FVC, FEV_1_/FVC) and proportions (sex, smoking) over three groups of individuals carrying the ε2 allele (ε2/ε2 or ε2/ε3 genotypes), the ε3/ε3 genotype, and the ε4 allele (ε4/ε4 or ε3/ε4 genotypes) were compared using one-way ANOVA with 2 degrees of freedom that does not require correction for multiple comparisons. Associations of the selected *APOE* genotypes with outcomes were characterized by a two-level mixed effects linear regression model (*lme4* package in R) with a random effect to adjust for the LLFS familial structure. Measurement of FEV_1_, FVC and FEV_1_/FVC were used in their natural scale because no noticeable skewness of their frequency distributions was detected. These statistical models were fitted considering the effect of the ε2 and ε4 alleles as compared to the common ε3/ε3 reference genotype.

Analyses of pooled samples and sex-stratified samples were performed. A test of multiplicative interactions was conducted to quantify differences in the effects between men and women. All statistical tests were adjusted for age, field center, family groups (i.e., probands, their offspring, and spouses) and sex, when applicable.

We investigated whether height, smoking, lipids, and body mass index (BMI) modulated associations of the *APOE* genotypes with FEV_1_, FVC and FEV_1_/FVC using models adjusted for each of these factors. BMI was calculated as weight (kg)/height (m)^2^. Standing height was measured using a Handi-stat set square (Perspective Enterprises, Portage, MI) to the nearest 0.1 cm. Weight was measured using an electronic digital scale (SECA 841, Hanover, MD) to the nearest 0.1 kg. Smoking was defined as current smokers. Total cholesterol, LDL- cholesterol (LDL-C), HDL-cholesterol (HDL-C), and triglycerides were measured in serum on a Roche/Modular P Chemistry Analyzer (Roche Diagnostics Corporation) using samples obtained after a 6-hour fast, which could occur during any time of the day. Total cholesterol was measured using a cholesterol oxidase enzymatic method and triglycerides were measured using a glycerol blanking enzymatic method (Roche Diagnostics, Indianapolis, IN). LDL-C was calculated using the Friedewald equation[39].

All analyses were conducted in R.

## Results

The 4,468 LLFS participants had a mean age of 70.06 ± 0.23 years and included 2,041 (44.82%) men. 636 subjects reported having lung disease, while 3,832 subjects were without lung disease. The frequencies of the selected three common *APOE* genotypes among men and women combined, as well as separately are shown in Table 1. The ε3/3 genotype was carried by 66.2% of LLFS participants (men and women combined), followed by ε4 and ε2 being carried by 17.8% and 16.0%, respectively. Carriers of the ε4 allele were the youngest and carriers of the ε2 allele were the oldest. The proportion of men was smallest among the ε3/ε3 carriers and largest among the ε2 carriers. Mean values of FEV_1_ and FVC were larger among ε4 carriers as compared to carriers of the ε3/ε3 genotype and ε2 allele when men and women were combined, as well as when stratified by sex. In contrast, the FEV_1_/FVC ratio was significantly lower among ε4 female carriers, whereas there was no significant difference in the FEV_1_/FVC ratio between *APOE* allele groups among men and women combined or among men.

**Table 1:** Baseline demographic characteristics and outcomes used in the analyses of the genotyped LLFS participants for men and women combined and separately.

Factor	*APOE*			*p-value (ANOVA)*
	ε2 = ε2/ε2 or ε2/ε3	ε3/ε3	ε4 = ε4/ε4 or ε3/ε4	
Men and Women				
N (%^†^)	715 (16.00)	2959 (66.23)	794 (17.77)	
Age, mean (SE), years	71.23 (0.63)	70.58 (0.29)	67.23 (0.49)	<0.001*
Sex, Male, N (%^†^)	345 (48.25)	1292 (43.66)	369 (46.47)	.053
Smoking, N (%^†^)	302 (42.30)	1238 (41.95)	358 (45.32)	.233
BMI, mean (SE), kg/m^2^	27.40 (10.01)	27.14 (9.43)	26.90 (9.75)	.151
CVD, N (%^†^)	189 (26.43)	789 (26.66)	185 (23.30)	.153
FEV_1_, mean (SE), [mL]	2398 (33)	2400 (16)	2571 (31)	<0.001*
FVC, mean (SE), [mL]	3121 (41)	3129 (20)	3366 (38)	<0.001*
FEV_1_/FVC, mean (SE) [%]	76.40 (0.28)	76.29 (0.14)	75.82 (0.27)	.261
Men				
N (%^†^)	345 (17.20)	1292 (64.41)	369 (18.39)	
Age, mean (SE), years	71.54 (0.88)	71.08 (0.43)	67.91 (0.71)	<0.001*
Smoking, N (%^†^)	166 (48.26)	639 (49.57)	185 (50.41)	.845
BMI, mean (SE), kg/m^2^	27.83 (8.33)	27.60 (7.98)	27.21 (7.92)	.124
CVD, N (%^†^)	100 (28.99)	406 (31.42)	106 (28.73)	.487
FEV_1_, mean (SE), [mL]	2806 (49)	2841 (25)	3040 (46)	<0.001*
FVC, mean (SE), [mL]	3664 (58)	3735 (29)	3989 (53)	<0.001*
FEV_1_/FVC, mean (SE) [%]	76.00 (0.44)	75.46 (0.23)	75.67 (0.39)	.553
Women				
N (%^†^)	370 (15.03)	1667 (67.71)	425 (17.26)	
Age, mean (SE), years	70.94 (0.90)	70.18 (0.40)	66.64 (0.68)	<0.001*
Smoking, N (%^†^)	136 (36.76)	599 (36.04)	173 (40.90)	.181
BMI, mean (SE), kg/m^2^	27.00 (11.31)	26.79 (10.38)	26.63 (11.10)	.651
CVD, N (%^†^)	89 (24.05)	383 (22.98)	79 (18.59)	.108
FEV_1_, mean (SE), [mL]	2018 (35)	2055 (17)	2169 (31)	.003*
FVC, mean (SE), [mL]	2615 (43)	2655 (20)	2834 (36)	<0.001*
FEV_1_/FVC, mean (SE) [%]	76.76 (0.36)	76.95 (0.17)	75.95 (0.38)	.044*

* denotes significant result (*p-value* < 0.05).

^†^ Percentage is within the considered group of the genotyped sample.

N denotes sample size.

Age is accessed at baseline.

Smoking status was defined as a cumulative smoking history of more than 100 cigarettes.

FEV_1_ denotes forced expiratory volume in 1 second.

FVC denotes forced vital capacity.

FEV_1_/FVC is the ratio (%) of FEV_1_ to FVC.

CVD denotes prevalence of cardiovascular diseases, which included coronary heart disease, heart failure, and stroke.

SE denotes standard error.

Baseline demographic characteristics and phenotypes for lung disease cases and controls without lung disease are given in Table 2.

P-values for the comparison of mean values (age, BMI, FEV_1_, FVC, FEV_1_/FVC) and proportions (sex, smoking) amongst three groups of individuals carrying the ε2 allele (ε2/ε2 or ε2/ε3 genotypes), the ε3/ε3 genotype, and the ε4 allele (ε4/ε4 or ε3/ε4 genotypes) were assessed by one-way ANOVA with 2 degrees of freedom.

**Table 2:** Associations of the *APOE* ε2 and ε4 alleles with FEV_1_, FVC, and FEV_1_/FVC of the genotyped LLFS participants for men and women combined and separately.

Trait	Effect allele	Men & Women			Men			Women		
		Beta	SE	P-value	Beta	SE	P-value	Beta	SE	P-value
FEV_1_	ε2	-11.24	22.40	.616	9.42	37.63	.802	-34.02	24.75	.169
	ε4	1.98	21.70	.927	39.50	37.97	.298	-12.34	23.46	.599
FVC	ε2	-34.41	23.09	.211	-44.68	46.19	.333	-34.37	30.95	.267
	ε4	23.09	26.08	.376	58.15	45.10	.197	19.93	29.19	.495
FEV_1_/FVC	ε2	0.12	0.32	.709	0.53	0.50	.288	-0.21	0.40	.595
	ε4	-0.70	0.30	.021*	-0.18	0.47	.703	-1.24	0.39	.001*

The ε3/ε3 genotype was considered as the reference.

SE denotes standard error.

Beta denotes the effect size.

* denotes significant result (*p-value* < 0.05).

Next, we assessed whether an association existed between spirometric values and *APOE* alleles in the LLFS participants. Table 2 shows that the ε4 allele is significantly associated with a 0.70% reduction in FEV_1_/FVC in the sample of men and women combined (p = 0.0214), thereby implying that the ε4 allele carriers have smaller FEV_1_/FVC values. Sex-stratified analysis showed that this effect is mostly attributed to women with a 1.24% reduction in the FEV_1_/FVC ratio (p = 0.0014). We also verified that the association with reduced FEV_1_/FVC in the sample of men and women combined did not reflect a potential bias related to population admixture (S1 Table). Further analyses showed that height, smoking, BMI (S2 Table), and lipids (S3 Table) did not mediate the associations of the *APOE* alleles with FEV_1_, FVC and FEV_1_/FVC. There were no significant interactions between the ε4 allele and BMI or smoking (S4 Table). There were also no significant interactions between the ε4 allele and lipids, except for HDL-C in men, in relationship to the FEV_1_/FVC ratio (S5 Table). Furthermore, the ε4 allele was significantly associated with FEV_1_/FVC in women with or without cardiovascular disease (S6 Table).

We next assessed whether this association persisted when we analyzed the large sub-group of 3,832 control subjects without lung disease (Table 3). This analysis showed a significant association between the ε4 allele and a 0.72% reduction in FEV_1_/FVC in the samples of men and women combined (p = 0.0164), as well as a 1.04% reduction in FEV_1_/FVC in women alone (p = 0.0078). We also assessed whether the association between the ε4 allele and FEV_1_/FVC in women was specifically related to aging (Table 4). This analysis showed that the association between the ε4 allele and FEV_1_/FVC was significant in long-living women (age 90.8 ± 6.9 years, mean ± standard deviation), but not in the spouses or children of long-living individuals, or in the spouses of offspring of long-living individuals. Similarly, the association between the ε4 allele and the FEV_1_/FVC was significant in LLFS women subjects ≥ 70 years, but not in LLFS women subjects < 70 years.

**Table 3:** Associations of the *APOE* ε2 and ε4 alleles with FEV_1_, FVC and FEV_1_/FVC in the sub-group of 3,832 genotyped LLFS participants without lung disease.

Trait	Effect allele	Men & Women			Men			Women		
		Beta	SE	P-value	Beta	SE	P-value	Beta	SE	P-value
FEV_1_	ε2	-5.44	22.92	.812	12.01	37.73	.750	-23.90	26.13	.360
	ε4	10.21	22.15	.645	44.26	38.25	.247	0.11	24.45	.996
FVC	ε2	-29.89	29.36	.309	-48.00	48.70	.324	-21.31	33.67	.527
	ε4	33.57	27.47	.222	70.34	46.89	.134	31.26	31.42	.320
FEV_1_/FVC	ε2	0.16	0.32	.605	0.45	0.48	.349	-0.03	0.42	.936
	ε4	-0.72	0.30	.016*	-0.46	0.46	.312	-1.04	0.39	.008*

The models were fitted with adjustments by age, sex, family groups, and field center.

Controls subjects without lung disease are defined in the Methods section.

The ε3/ε3 genotype was considered as the reference.

* denotes significant result (*p-value* < 0.05).

**Table 4:** Associations of the *APOE* ε2 and ε4 alleles with FEV_1_/FVC in different age groups of LLFS participants.

Trait	Effect allele	Men & Women				Men				Women			
		N	Beta	SE	P-value	N	Beta	SE	P-value	N	Beta	SE	P-value
FEV_1_/FVC LL individuals	ε2	968	0.41	0.73	.575	468	0.34	1.04	.745	500	0.48	1.02	.639
	ε4	907	-1.47	0.87	.098	437	0.05	1.21	.967	470	-2.98	1.23	.027*
FEV_1_/FVC LL spouses	ε2	112	1.25	1.76	.513	23	2.75	3.11	.388	89	1.76	2.11	.466
	ε4	121	-3.08	1.63	.200	26	-0.30	2.62	.909	95	-4.05	2.01	.294
FEV_1_/FVC LL offspring	ε2	1696	-0.27	0.36	.450	713	0.31	0.60	.612	983	-0.59	0.42	.169
	ε4	1739	-0.36	0.34	.286	731	-0.27	0.54	.623	1008	-0.55	0.43	.206
FEV_1_/FVC Offspring spouses	ε2	533	0.67	0.85	.436	277	1.40	1.27	.282	256	-0.43	1.07	.694
	ε4	630	-0.55	0.62	.378	315	-0.37	1.01	.714	315	-0.87	0.71	.225
FEV_1_/FVC age < 70	ε2	1914	-0.21	0.35	.554	826	0.43	0.57	.450	1088	-0.58	0.42	.169
	ε4	2051	-0.30	0.31	.340	886	-0.24	0.50	.629	1165	-0.39	0.39	.318
FEV_1_/FVC age ≥ 70	ε2	1395	0.59	0.58	.307	655	0.89	0.86	.305	740	0.34	0.77	.657
	ε4	1346	-1.76	0.63	.006*	623	-0.06	0.95	.947	723	-3.19	0.84	<0.001*

LL indicates long-living individuals. Mean age and standard deviation (SD) for LL individuals were: 90.4 ± 6.4 years (men and women combined), 89.8 ± 5.7 years (men) and 90.8 ± 6.9 years (women). Mean age and SD for spouses of LL individuals were: 83.1 ± 7.0 years (men and women combined), 85.3 ± 5.5 years (men) and 82.5 ± 7.3 years (women). Mean age and SD for offspring of LL individuals were: 60.5 ± 8.2 years (men and women combined), 60.6 ± 8.2 years (men) and 60.5 ± 8.2 years (women). Mean age and SD for spouses of offspring were: 60.9 ± 8.6 years (men and women combined), 63.1 ± 7.9 years (men) and 58.4 ± 8.7 years (women).

LLFS participants were also divided into subjects < or ≥ 70 years of age.

* denotes significant result (*p-value* < 0.05).

Lastly, an analysis of interactions showed that sex modified the effect of the ε4 allele on FEV_1_, FVC, and FEV_1_/FVC in the LLFS participants (Table 5). The interaction between the ε4 allele and sex gained nominal significance (p = 0.045) for FEV_1_/FVC, while there was a trend for FEV_1_ (p = 0.060). These results suggest a sexual dimorphism in the associations between the *APOE* ε4 allele and FEV_1_ and FEV_1_/FVC.

**Table 5:** Associations of the *APOE* ε2 and ε4 alleles with FEV_1_, FVC, and FEV_1_/FVC and their multiplicative interactions with sex in the genotyped LLFS participants.

Trait	apoE	apoE			apoE×sex		
		Beta	SE	P-value	Beta	SE	P-value
FEV_1_	ε2	-29.44	30.83	.340	13.36	43.82	.761
	ε4	-33.60	29.03	.247	79.36	42.20	.060
FVC	ε2	-28.98	36.97	.433	-12.70	52.56	.810
	ε4	-8.92	34.56	.796	69.46	50.21	.167
FEV_1_/FVC	ε2	-0.20	0.43	.643	0.70	0.62	.258
	ε4	-1.22	0.40	.003*	1.18	0.59	.045*

The models with interactions considered men as a reference category.

The ε3/ε3 genotype was considered as the reference.

* denotes significant result (*p-value* < 0.05).

## Discussion

The human *APOE* gene is polymorphic and encodes three distinct protein isoforms, APOE2, APOE3, and APOE4, which differ by single amino acid substitutions at amino acids 112 and 158[1, 3]. APOE3 is the most common isoform and contains a cysteine at position 112 and an arginine at position 158, whereas APOE2 contains two cysteines and APOE4 has two arginines[1]. Although these polymorphisms do not involve the receptor- or lipid-binding domains of APOE, they significantly alter its structural and functional properties. APOE2 has markedly impaired LDLR binding capabilities, which is less than 2% of APOE3 and APOE4[2]. The APOE4 isoform has normal LDLR binding, but has altered lipid binding characteristics, with preferential binding to triglyceride-rich VLDL and chylomicron remnants instead of phospholipid-rich high-density lipoproteins[2].

The *APOE ε4* allele is the minor allele in modern humans and is a major genetic risk factor for Alzheimer’s disease[1, 2, 40]. The APOE ε4 allele also increases atherosclerosis risk and decreases lifespan[2, 40]. *APOE* ε*4* is the ancestral *APOE* allele, as it is present in all great apes, while the shift to the *APOE* ε*3* and *APOE* ε*2* alleles may have reflected selective pressure from infectious diseases[2]. Consistent with this, the human *APOE* ε*4* allele modifies host responses to infection, such as the human immunodeficiency virus[2, 40]. Transgenic knock-in mice expressing the human *APOE* ε*4* allele have increased morbidity and mortality in experimental sepsis models[7, 41]. Similarly, human subjects heterozygous for *APOE* ε3/ε4 have increased innate immune responses to LPS as compared to individuals with the *APOE* ε3/ε3 genotype[41]. Furthermore, the *APOE* ε*4* allele has been associated with increased coagulation system failure in European Americans with severe sepsis[41]. Susceptibility to infection by the intracellular bacterial pathogen, *Chlamydia pneumoniae*, may also be enhanced by the *APOE* ε*4* allele by a mechanism involving increased attachment to host cells[42].

The *APOE* alleles, however, are not known to modulate pulmonary function in human subjects and have not been linked to pulmonary function in genome-wide association studies[43–51]. Although a prior genome-wide association study of LLFS participants identified a linkage peak on chromosome 2 for FEV_1_/FVC, the *APOE* ε2, ε3 and ε4 alleles were not included in that dataset[48]. Here, we show that the mean FEV_1_/FVC ratio was lower among women with the ε4 allele as compared to women with either the ε3/3 genotype or the ε2 allele. In addition, carriage of the *APOE* ε*4* allele in the LLFS participants was associated with the FEV_1_/FVC ratio, which implied a smaller value, as compared to carriers of the *APOE* ε3 allele. Although a reduction in the FEV_1_/FVC ratio could reflect the presence of airflow obstruction, both the FVC and FEV_1_ were increased in carriers of the *APOE* ε*4* allele as compared to carriers of the ε3/3 genotype or the ε2 allele. This suggests that the reduction in the FEV_1_/FVC ratio in carriers of the *APOE* ε*4* allele might instead represent modified lung physiology reflected by a proportionally larger FVC than FEV_1_. For example, dysanaptic or unequal lung growth has been reported to occur in teenagers, where growth of the airways, as reflected by the FEV_1_, relative to lung volumes, as reflected by the FVC, occurs more slowly in girls than boys[52, 53]. Furthermore, dysanaptic lung growth has been reported in the setting of chronic hypoxia and post-infectious bronchiolitis obliterans, which results in an decrease in the FEV_1_/FVC ratio[53, 54]. Since the reduction in FEV_1_/FVC in carriers of the *APOE* ε*4* allele was specific for individuals over 70 years of age, we believe that it is unlikely that dysanaptic lung development is the explanation for this finding. The FEV_1_/FVC ratio is well-known to decrease with advancing age, which suggests that the reduction in FEV_1_/FVC among carriers of the *APOE* ε*4* allele might instead reflect modified lung aging in older individuals[55–58]. This finding will need to be confirmed by future studies that assess whether the reduction in FEV_1_/FVC among carriers of the *APOE* ε*4* allele is limited to older individuals, in which case it would not be generalizable to younger study populations. Similarly, future studies would be required to assess whether this finding is limited to individuals with exceptional familial longevity or is also generalizable to populations with normal longevity.

The association between the *APOE* ε*4* allele and a lower FEV_1_/FVC ratio persisted when the sub-group of LLFS women without lung disease was analyzed. To the best of our knowledge, this demonstrates for the first time that an association exists between the polymorphic *APOE* alleles and lung function in human subjects. Although the effect size on FEV_1_/FVC was modest, the effect of an individual allele on a complex genetic trait is often relatively small. In addition, since the effect was present in individuals without lung disease, a large magnitude of change was not expected. Furthermore, our results suggest that a link might exist between the *APOE* ε*4* allele, female sex, and a lower FEV_1_/FVC ratio in older individuals. This may provide a new insight regarding how the polymorphic *APOE* alleles might influence human lung physiology in a gender-specific fashion during aging.

These data also raise the hypothesis that the *APOE* alleles might modify disease pathogenesis or severity in obstructive airway diseases, such as asthma, COPD, or asthma-COPD overlap syndrome. In a prior study we showed that serum apoE levels were similar in normals and asthmatics and were not correlated with FEV_1_, however, subjects were not stratified by *APOE* genotype[59]. A limitation of this current study is that we were not able to assess if the *APOE* alleles modified pulmonary function in subjects with lung disease. We did not include a sub-group analysis of cases with lung disease for several reasons. First, lung disease was self-reported by participants at baseline and during follow up, but the diagnosis of lung disease was not confirmed. Second, because the types of lung disease were heterogeneous regarding diagnoses, severity, and duration, and the number of cases with lung disease was small, an analysis of this sub-group would likely not have sufficient power to identify an association between the *APOE* alleles and pulmonary function. Third, a subset of the cases with lung disease were utilizing lung disease medications, such as bronchodilators and corticosteroids, which could have modified their pulmonary function results. Therefore, additional studies will be required to assess whether the *APOE* ε*4* allele is associated with modified pulmonary function in individuals with lung disease. Another limitation of this study is that LLFS participants were selected based upon the trait of exceptional familial longevity. Therefore, additional studies will be needed to assess whether the association between the FEV_1_/FVC ratio and the *APOE* ε*4* allele in older women is also present in individuals who do not have long-living families.

The mechanisms by which the *APOE* ε*4* allele might be associated with a relative reduction in airflow in women without lung disease are not known. Prior murine studies have identified that the APOE/LDLR pathway is present in the lung, with the LDLR being expressed by ciliated airway epithelial cells where it could potentially modify airway physiology[10]. Furthermore, studies using *Apoe*- and *Ldlr*-deficient mice have shown that these genes participate in developmental alveologenesis in a sexually dimorphic fashion[13, 14]. In addition, *Apoe*- and *Ldlr*-deficient mice that have been sensitized and challenged with house dust mite have abnormal airway function with increased mucous cell metaplasia and airway hyperreactivity to methacholine[10]. The *APOE* ε*4* allele has also been associated with enhanced risk and accelerated decline in cognitive function secondary to Alzheimer’s disease in women as compared to men[60]. The *APOE* ε*4* allele was also highly significantly associated with shorter lifespan in women as compared to men in the LLFS study and the Framingham Heart Study[61]. Furthermore, prior studies have supported the concept of sexual dimorphism of the ε*4* allele in relationship to *APOE*-related mortality and heart disease[62–65]. Gender-specific effects of the *APOE* gene might be attributed to differential hormonal and insulin regulation in each sex[62]. Collectively, these findings provide additional evidence to support a gender-specific effect of the ε*4* allele on the FEV_1_/FVC ratio.

Based upon differences in height and lung volume, absolute values for FEV_1_ and FVC are known to be lower in adult women than adult men[66, 67]. Consistent with this, we found that the absolute values for FEV_1_ and FVC were significantly lower in women than men, irrespective of whether they carried the APOE ε2, ε3, or ε4 allele, as evidenced by well-separated 95% confidence intervals for FEV_1_ and FVC measured in men and women as +/- 1.96*SE (Table 1). In contrast, the FEV_1_/FVC ratio was not different between men and women when the sample population was not stratified by age. Therefore, we believe that our finding of a significant reduction in the FEV_1_/FVC ratio limited to the subset of older women greater or equal to 70 years of age who carried the APOE ε4 allele did not merely reflect a compounding effect of genotype in individuals who already had lower values by virtue of sex.

In conclusion, we have identified that carriage of the *APOE* ε*4* allele was associated with a decrease in the FEV_1_/FVC ratio as compared to carriers of the *APOE* ε*3* allele among women without lung disease who participated in the LLFS. This suggests that carriage of the *APOE* ε*4* allele may be linked to modified lung physiology in a sexually dimorphic fashion in older individuals.

## Supporting information

S1 Table The role of potential population structure in the associations of the *APOE* ε2 and ε4 alleles with FEV_1_, FVC and FEV_1_/FVC in the genotyped LLFS participants. (DOCX)

S2 Table The role of height, smoking, and BMI in the associations of the *APOE* ε2 and ε4 alleles with FEV_1_, FVC and FEV_1_/FVC in the genotyped LLFS participants. (DOCX)

S3 Table The role of lipids in the associations of the *APOE* ε2 and ε4 alleles with FEV_1_, FVC and FEV_1_/FVC in the genotyped LLFS participants. (DOCX)

S4 Table Interactions of the *APOE* ε2 and ε4 alleles with smoking and BMI in the relationship to FEV_1_/FVC. (DOCX)

S5 Table Interactions of the *APOE* ε2 and ε4 alleles with lipids in the relationship to FEV_1_/FVC. (DOCX)

S6 Table Associations of the *APOE* ε2 and ε4 alleles with FEV_1_/FVC in individuals with and without cardiovascular disease (CVD). (DOCX)
