# Supplementary material for: The APOE ε4 allele is associated with a reduction in FEV1/FVC in women: A cross-sectional analysis of the Long Life Family Study
Source: PLoS One. 2018 Nov 9;13(11):e0206873. doi: 10.1371/journal.pone.0206873 (PMC6226172; doi:10.1371/journal.pone.0206873)
Supplement: S1 Table — (DOCX) [file pone.0206873.s001.docx]

**Supplemental Table 1.** The role of potential population structure in the associations of the *APOE* ε2 and ε4 alleles with FEV_1_, FVC and FEV_1_/FVC in the genotyped LLFS participants.

| Trait | Effect  allele | N | Men & Women, Model 1 | | | Men & Women, Model 2 | | |
| --- | --- | --- | --- | --- | --- | --- | --- | --- |
|  |  |  | Beta | SE | P-value | Beta | SE | P-value |
| FEV_1_ | ε2 | 3,251 | -15.75 | 22.53 | .484 | -14.03 | 22.53 | .533 |
|  | ε4 | 3,342 | -2.79 | 21.78 | .898 | -3.65 | 21.78 | .867 |
| FVC | ε2 | 3,251 | -24.27 | 26.94 | .368 | -21.96 | 26.94 | .415 |
|  | ε4 | 3,342 | 15.83 | 25.94 | .542 | 15.24 | 25.94 | .557 |
| FEV_1_/FVC | ε2 | 3,251 | 0.09 | 0.32 | .785 | 0.08 | 0.32 | .808 |
|  | ε4 | 3,342 | -0.72 | 0.30 | .018^*^ | -0.73 | 0.30 | .016^*^ |

The ε3/ε3 genotype was considered as the reference.

Model 1: basic adjustments (age, sex, family groups, and field center) only.

Model 2: basic adjustments + first five principal components.

The models were fitted for the sample of men and women combined with no stratification by lung disease status. We excluded individuals with missing information on principal components to match the sample sizes in models 1 and 2. Because of this exclusion, the results in this table for model 1 are slightly different from those in Table 2.

^*^ denotes significant result (*p-value* < 0.05).
