# Supplementary material for: The APOE ε4 allele is associated with a reduction in FEV1/FVC in women: A cross-sectional analysis of the Long Life Family Study
Source: PLoS One. 2018 Nov 9;13(11):e0206873. doi: 10.1371/journal.pone.0206873 (PMC6226172; doi:10.1371/journal.pone.0206873)
Supplement: S3 Table — (DOCX) [file pone.0206873.s003.docx]

**Supplemental** **Table 3**. The role of lipids in the associations of the *APOE* ε2 and ε4 alleles with FEV_1_, FVC and FEV_1_/FVC in the genotyped LLFS participants.

| Trait | Effect  allele | N | Men & Women, Model 1 | | | Men & Women, Model 2 | | |
| --- | --- | --- | --- | --- | --- | --- | --- | --- |
|  |  |  | Beta | SE | P-value | Beta | SE | P-value |
| Total cholesterol (TC) | | | | | | | | |
| FEV_1_ | ε2 | 3,230 | -9.17 | 22.57 | .685 | 1.34 | 22.67 | .953 |
|  | ε4 | 3,320 | 3.80 | 21.83 | .862 | 2.18 | 21.76 | .920 |
| FVC | ε2 | 3,239 | -32.10 | 27.73 | .247 | -18.01 | 27.84 | .518 |
|  | ε4 | 3,325 | 26.97 | 26.23 | .304 | 25.14 | 26.14 | .336 |
| FEV_1_/FVC | ε2 | 3,230 | 0.13 | 0.32 | .679 | 0.14 | 0.33 | .678 |
|  | ε4 | 3,320 | -0.72 | 0.30 | .018^*^ | -0.73 | 0.30 | .017^*^ |
| Low-density lipoprotein cholesterol (LDL-C) | | | | | | | | |
| FEV_1_ | ε2 | 3,202 | -10.48 | 22.64 | .643 | 7.41 | 22.92 | .747 |
|  | ε4 | 3,294 | 9.22 | 21.80 | .672 | 4.92 | 21.71 | .821 |
| FVC | ε2 | 3,211 | -33.41 | 27.85 | .230 | -13.77 | 28.20 | .625 |
|  | ε4 | 3,299 | 33.97 | 26.21 | .195 | 29.41 | 26.13 | .260 |
| FEV_1_/FVC | ε2 | 3,202 | 0.13 | 0.33 | .685 | 0.24 | 0.33 | .477 |
|  | ε4 | 3,294 | -0.72 | 0.31 | .018^*^ | -0.75 | 0.31 | .014^*^ |
| High-density lipoprotein cholesterol (HDL-C) | | | | | | | | |
| FEV_1_ | ε2 | 3,229 | -9.05 | 22.57 | .688 | -9.98 | 22.56 | .658 |
|  | ε4 | 3,319 | 3.90 | 21.83 | .858 | 5.63 | 21.85 | .796 |
| FVC | ε2 | 3,238 | -31.82 | 27.73 | .251 | -34.94 | 27.59 | .205 |
|  | ε4 | 3,324 | 27.19 | 26.22 | .300 | 33.68 | 26.16 | .198 |
| FEV_1_/FVC | ε2 | 3,229 | 0.13 | 0.32 | .687 | 0.18 | 0.32 | .583 |
|  | ε4 | 3,319 | -0.73 | 0.30 | .017^*^ | -0.81 | 0.30 | .008^*^ |
| Triglycerides (TG) | | | | | | | | |
| FEV_1_ | ε2 | 3,230 | -9.17 | 22.57 | .685 | -6.22 | 22.59 | .783 |
|  | ε4 | 3,320 | 3.80 | 21.83 | .862 | 5.27 | 21.81 | .809 |
| FVC | ε2 | 3,239 | -32.10 | 27.73 | .247 | -25.71 | 27.71 | .357 |
|  | ε4 | 3,325 | 26.97 | 26.23 | .304 | 26.14 | 26.14 | .248 |
| FEV_1_/FVC | ε2 | 3,230 | 0.13 | 0.32 | .679 | 0.06 | 0.32 | .841 |
|  | ε4 | 3,320 | -0.72 | 0.30 | .018^*^ | -0.74 | 0.30 | .015^*^ |

The ε3/ε3 genotype was considered as the reference.

Model 1: basic adjustments (age, sex, family groups, and field center) only.

Model 2: basic adjustments + TC, LDL-C, HDL-C, or TG.

The models were fitted for the sample of men and women combined with no stratification by lung disease status. We excluded individuals with missing information on TC, LDL-C, HDL-C, or TG to match the sample sizes in models 1 and 2. Because of this exclusion, the results in this table for model 1 are slightly different from those in Table 2.

The results are qualitatively the same for each sex.

^*^ denotes significant result (*p-value* < 0.05).
