# Supplementary material for: The APOE ε4 allele is associated with a reduction in FEV1/FVC in women: A cross-sectional analysis of the Long Life Family Study
Source: PLoS One. 2018 Nov 9;13(11):e0206873. doi: 10.1371/journal.pone.0206873 (PMC6226172; doi:10.1371/journal.pone.0206873)
Supplement: S4 Table — (DOCX) [file pone.0206873.s004.docx]

**Supplemental Table 4**. Interactions of the *APOE* ε2 and ε4 alleles with smoking and BMI in the relationship to FEV_1_/FVC.

| Trait | Effect  allele | Men & Women | | | Men | | | Women | | |
| --- | --- | --- | --- | --- | --- | --- | --- | --- | --- | --- |
|  |  | Beta | SE | P-value | Beta | SE | P-value | Beta | SE | P-value |
| FEV_1_/FVC  No interactions | ε2 | 0.14 | 0.32 | .670 | 0.56 | 0.50 | .260 | -0.21 | 0.40 | .608 |
|  | ε4 | -0.69 | 0.30 | .024^*^ | -0.13 | 0.47 | .780 | -1.23 | 0.39 | .002^*^ |
| FEV_1_/FVC  Interaction  with smoking | ε2 | 0.14 | 0.48 | .780 | 0.24 | 0.70 | .730 | 0.02 | 0.65 | .970 |
|  | ε2*smoking | 0.004 | 0.62 | .990 | 0.55 | 0.96 | .570 | -0.34 | 0.81 | .680 |
|  | ε4 | -0.60 | 0.45 | .180 | 0.42 | 0.65 | .520 | -1.92 | 0.61 | .002^*^ |
|  | ε4*smoking | -0.11 | 0.59 | .850 | -1.10 | 0.92 | .230 | 1.20 | 0.78 | .120 |
| FEV_1_/FVC  Interaction  with BMI | ε2 | 0.06 | 0.37 | .879 | 0.18 | 0.58 | .757 | -0.07 | 0.46 | .883 |
|  | ε2*BMI | 0.18 | 0.71 | .795 | 1.23 | 1.09 | .259 | -0.66 | 0.92 | .475 |
|  | ε4 | -0.96 | 0.34 | .005^*^ | -0.27 | 0.53 | .609 | -1.58 | 0.43 | <0.001^*^ |
|  | ε4*BMI | 1.38 | 0.72 | .055 | 0.84 | 1.13 | .453 | 1.69 | 0.93 | .070 |

The ε3/ε3 genotype was considered as the reference.

Smoking status was defined as a cumulative smoking history of more than 100 cigarettes.

Body mass index (BMI) was dichotomized according to cut off between overweight and obesity, i.e., BMI < 30 kg/m^2^ and BMI > 30 kg/m^2^.

^*^ denotes significant result (*p-value* < 0.05).
