# Supplementary material for: The APOE ε4 allele is associated with a reduction in FEV1/FVC in women: A cross-sectional analysis of the Long Life Family Study
Source: PLoS One. 2018 Nov 9;13(11):e0206873. doi: 10.1371/journal.pone.0206873 (PMC6226172; doi:10.1371/journal.pone.0206873)
Supplement: S5 Table — (DOCX) [file pone.0206873.s005.docx]

**Supplemental Table 5**. Interactions of the *APOE* ε2 and ε4 alleles with lipids in the relationship to FEV_1_/FVC.

| Trait | Effect  allele | Men & Women | | | Men | | | Women | | |
| --- | --- | --- | --- | --- | --- | --- | --- | --- | --- | --- |
|  |  | Beta | SE | P-value | Beta | SE | P-value | Beta | SE | P-value |
| FEV_1_/FVC  No interactions | ε2 | 0.13 | 0.32 | .686 | 0.53 | 0.50 | .284 | -0.20 | 0.40 | .621 |
|  | ε4 | -0.77 | 0.30 | .024^*^ | -0.15 | 0.47 | .750 | -1.21 | 0.39 | .003^*^ |
| FEV_1_/FVC  Interaction  with TC | ε2 | 0.43 | 0.40 | .293 | 0.64 | 0.59 | .281 | 0.18 | 0.55 | .741 |
|  | ε2*TC | -0.75 | 0.64 | .231 | -0.60 | 1.08 | .582 | -0.77 | 0.80 | .335 |
|  | ε4 | -0.92 | 0.43 | .035^*^ | -0.55 | 0.62 | .374 | -1.52 | 0.61 | .014^*^ |
|  | ε4*TC | 0.44 | 0.59 | .455 | 0.92 | 0.94 | .325 | 0.49 | 0.78 | .532 |
| FEV_1_/FVC  Interaction  with LDL-C | ε2 | 0.49 | 0.47 | .299 | 0.67 | 0.70 | .343 | 0.28 | 0.62 | .654 |
|  | ε2*LDL-C | -0.58 | 0.63 | .356 | -0.30 | 0.98 | .758 | -0.72 | 0.81 | .376 |
|  | ε4 | -1.01 | 0.55 | .066 | -0.47 | 0.79 | .546 | -1.83 | 0.77 | .018^*^ |
|  | ε4*LDLC | 0.46 | 0.65 | .475 | 0.50 | 0.97 | .606 | 0.81 | 0.88 | .362 |
| FEV_1_/FVC  Interaction  with HDL-C | ε2 | -0.61 | 0.77 | .430 | -1.01 | 1.03 | .327 | -1.41 | 0.82 | .089 |
|  | ε2*HDLC | 0.87 | 0.84 | .300 | 2.02 | 1.16 | .081 | 1.54 | 0.93 | .097 |
|  | ε4 | -1.59 | 0.74 | .032^*^ | -1.86 | 0.92 | .046^*^ | -1.37 | 0.79 | .086 |
|  | ε4*HDLC | 1.03 | 0.80 | .198 | 2.21 | 1.07 | .039^*^ | 0.15 | 0.90 | .867 |
| FEV_1_/FVC  Interaction  with TG | ε2 | 0.01 | 0.36 | .970 | 0.39 | 0.56 | .491 | -0.21 | 0.45 | .646 |
|  | ε2*TG | 0.24 | 0.73 | .740 | 0.29 | 1.14 | .803 | -0.09 | 0.96 | .925 |
|  | ε4 | -0.76 | 0.33 | .023^*^ | 0.04 | 0.53 | .946 | -1.42 | 0.42 | <.001^*^ |
|  | ε4*TG | 0.36 | 0.77 | .639 | -1.00 | 1.13 | .379 | 1.46 | 1.07 | .173 |

The ε3/ε3 genotype was considered as the reference.

Total cholesterol (TC) was dichotomized according to cut off between normal and not normal levels of TC, i.e., TC < 200 mg/dL and TC > 200 mg/dL.

Low-density lipoprotein cholesterol (LDL-C) was dichotomized according to cut off between normal and not normal levels of LDLC, i.e., LDL-C < 100 mg/dL and LDL-C > 100 mg/dL.

High-density lipoprotein cholesterol (HDL-C) was dichotomized according to cut off between normal and not normal sex-specific levels of HDL-C, i.e., HDL-C < 40 mg/dL and HDL-C > 40 mg/dL for men and HDL-C < 50 mg/dL and HDL-C > 50 mg/dL for women. For the sample of men and women combined, we used the same dichotomization as for men.

Triglycerides (TG) was dichotomized according to cut off between normal and not normal levels of TG, i.e., TG < 150 mg/dL and TG > 150 mg/dL.

^*^ denotes significant result (*p-value* < 0.05).
