# Supplementary material for: The APOE ε4 allele is associated with a reduction in FEV1/FVC in women: A cross-sectional analysis of the Long Life Family Study
Source: PLoS One. 2018 Nov 9;13(11):e0206873. doi: 10.1371/journal.pone.0206873 (PMC6226172; doi:10.1371/journal.pone.0206873)
Supplement: S6 Table — (DOCX) [file pone.0206873.s006.docx]

**Supplemental Table 6**. Associations of the *APOE* ε2 and ε4 alleles with FEV_1_/FVC in individuals with and without cardiovascular disease (CVD).

| Trait | Effect  allele | Men & Women | | | Men | | | Women | | |
| --- | --- | --- | --- | --- | --- | --- | --- | --- | --- | --- |
|  |  | Beta | SE | P-value | Beta | SE | P-value | Beta | SE | P-value |
| FEV_1_/FVC  no CVD | ε2 | 0.01 | 0.33 | .976 | 0.23 | 0.54 | .668 | -0.15 | 0.41 | .721 |
|  | ε4 | -0.73 | 0.31 | .021^*^ | -0.54 | 0.51 | .293 | -0.90 | 0.39 | .022^*^ |
| FEV_1_/FVC  CVD cases | ε2 | 0.70 | 0.77 | .368 | 1.13 | 1.06 | .294 | -0.24 | 1.09 | .825 |
|  | ε4 | -0.76 | 0.77 | .326 | 0.79 | 1.04 | .452 | -2.91 | 1.14 | .016^*^ |

CVD denotes prevalence of cardiovascular diseases, which included coronary heart disease, heart failure, and stroke.

^*^ denotes significant result (*p-value* < 0.05).
